# Mathematical Models of Parathyroid Gland Biology: Complexity and Clinical Use

**DOI:** 10.3389/fneph.2022.893391

**Published:** 2022-05-18

**Authors:** Gudrun Schappacher-Tilp, Peter Kotanko, Markus Pirklbauer

**Affiliations:** ^1^Department of Electronic Engineering, University of Applied Science FH Joanneum, Graz, Austria; ^2^Institute for Mathematics and Scientific Computing, University of Graz, Graz, Austria; ^3^Renal Research Institute New York, New York, NY, United States; ^4^Icahn School of Medicine at Mount Sinai, New York, NY, United States; ^5^Department of Internal Medicine IV - Nephrology and Hypertension, Medical University Innsbruck, Innsbruck, Austria

**Keywords:** Parathyroid gland biology, parathyroid hormone, mathematical models, predictions, chronic kidney disease - mineral and bone disorder (CKD-MDB)

## Abstract

Altered parathyroid gland biology is a major driver of chronic kidney disease-mineral bone disorder (CKD-MBD) in patients with chronic kidney disease. CKD-MBD is associated with a high risk of vascular calcification and cardiovascular events. A hallmark of CKD-MBD is secondary hyperparathyroidism with increased parathyroid hormone (PTH) synthesis and release and reduced expression of calcium-sensing receptors on the surface of parathyroid cells and eventually hyperplasia of parathyroid gland cells. The KDIGO guidelines strongly recommend the control of PTH in hemodialysis patients. Due to the complexity of parathyroid gland biology, mathematical models have been employed to study the interaction of PTH regulators and PTH plasma concentrations. Here, we present an overview of various model approaches and discuss the impact of different model structures and complexities on the clinical use of these models.

## 1 Introduction

Mathematical modeling of physiological processes has witnessed tremendous development in recent years. Mathematical models have become a vital research area with ever-evolving computational resources, data analytic tools and techniques, and experimental setups. In fact, in PubMed, the search term “mathematical model(l)ing” or “mathematical model” yields 1.592.770 results since 2000. That number is more than four-fold the number of results gained with the same research term but restricted to 1933 -1999.^^1^^

Mathematical modeling is the act of translating a hypothesis into the mathematical framework, analyzing the hypothesis within this framework, and translating the results back to the real world by making quantitative predictions that can be compared to experimental or clinical results. Mathematical models are vital for analysing and understanding the relationship between complex system components. Structural mathematical models can provide mechanistic insight into complex biological systems which show non-linear behavior. Individualized mathematical models can be used to predict the efficacy of treatment options, such as the administration of calcimimetics.

A crucial part of the modeling process is the choice of the components that should be part of the models. This choice depends on the complexity of the underlying hypothesis, the desired accuracy in model predictions, the robustness of model predictions, the availability of experimental or clinical data, and sufficient computing time. There is always a trade-off between model complexity and validation in mathematical modeling of physiological processes. While highly complex models can take complex interactions of physiological processes into account and may even help develop a new hypothesis, validation of these models is often limited by the quality, quantity, and heterogeneity of data. Besides validation, the robustness of model results and predictions is a well-known problem – both decrease with increasing model complexity. Once a model has been chosen, the next step is model parametrization. More often than not, model parameters have to be chosen indirectly by comparing experimental or clinical measurements and model predictions.

The clinical use of mathematical models strongly depends on the predictive power of these models. Here, we will discuss mathematical parathyroid gland (PTG) biology models that focus on the patient cohort of chronic kidney disease (CKD) patients. The parathyroid gland produces, stores, and eventually releases the parathyroid hormone (PTH) (1). PTH is the key endocrine regulator of calcium homeostasis. It has direct and indirect effects on phosphate and Ca^2+^concentrations by its actions in the kidneys and the bone. Specifically, PTH facilitates bone resorption and thereby, the mobilization of calcium and phosphate from the bone. It has a phosphaturic effect (2, 3) while increasing resorption of calcium within the kidney (4, 5). Finally, PTH stimulates the synthesis of the steroid hormone calcitriol, the biologically active form of vitamin D, *via* the activation of 1α-hydroxylase in the kidneys (6, 7). Calcitriol enhances the active transport of calcium and phosphate in the intestine (8–10). The activity of PTG cells is mainly governed by the calcium-sensing receptors surfacing the cells (11, 12). Upon activation by ionized calcium (Ca^2+^), the downstream signaling of this G-coupled protein suppresses PTH release. If there is a drop in Ca^2+^, the PTG reacts quickly by releasing pre-formed and stored PTH (13). The PTG biology is complex as the cells can adapt (1). The intracellular degradation rate can be decreased within minutes (14, 15), and the production can be increased within minutes to hours (16). If the cells are signaled that there is still high demand for PTH, the proliferation rate will increase, eventually resulting in hyperplasia (17–19). Another receptor standing out in PTG regulation is the vitamin D receptor within the PTG cells. There is a positive feedback loop between the vitamin D receptor (VDR) and the CaSR (19–21), which is known to be diminished by high phosphate levels (22). Recently, it has been shown that phosphate also has a direct stimulating effect on PTH release (23).

The control of PTH is of high priority for precision medicine approaches in HD patients. Specifically, KDIGO guidelines recommend iPTH target levels of 2-9 times the upper limit of normal for the assay, reflecting the increased risk with low PTH levels associated with adynamic bone disease and high PTH levels associated with high bone turnover disease (24). Ambiguous clinical situations reflect the complex biology of PTG during CKD progression. The heterogeneous clinical phenotypes include, for example, responders and nonresponders to PTH lowering drugs in incident dialysis patients with mildly elevated iPTH levels. Recently identified polymorphisms in genes of the CaSR and the VDR may partly account for the heterogeneity among HD patients (25).

With mathematical models, we can study many aspects of PTG biology. Specifically, mathematical models are valuable for breaking down the multidimensional cascade of PTG adaptation processes, thereby enabling the detailed study of subsystems. Personalized models of PTG biology could predict the efficacy of treatment and thereby help the physician to find the optimal treatment strategy. Generally, a personalized PTG model requires two essential components, a generic (i.e., non-personalized) model of PTG biology and clinical data to estimate patient-specific model parameter. Ideally, the clinical data is readily available. The clinical use of these models highly depends on the aim, the application, and the quality and quantity of data needed for the parameter estimation. Here, we will discuss the different modeling approaches and their possible benefits for clinical use.

## 2 Mathematical Models of Parathyroid Gland Biology

### 2.1 Ca-Dependent PTH Release

The simplest mechanism of interest in PTG biology is PTH concentrations’ functional response to Ca^2+^. The PTG responds to acute changes in plasma Ca^2+^concentrations within minutes (11). There is a steep slope in the relationship between plasma ca and PTH concentrations near the individual optimal Ca^2+^concentration indicating not only a swift but also a profound response in PTH secretion rate. It is suggested that CaSR signaling is amplified by either cooperative binding of Ca^2+^or cooperative intracellular signaling (26, 27). The time scale of the acute response is significantly faster than the time scale of other adaptation mechanisms, such as a decrease in intracellular PTH degradation. Therefore, if the only interest lies in the acute response of PTH to changes in Ca^2+^, a simple model reflecting the changes in PTH secretion rate is sufficient.

The simplest model of PTH response to Ca^2+^has the form


(1) $$\frac{dPTH}{dt}=k_{rel}(Ca)-k_{cl}PTH(t)$$

The release rate is a sigmoidal function of Ca^2+^ (11), e.g.:


(2) $$k_{rel}(Ca)=r_{basal}+\frac{r_{max}-r_{basal}}{1+{\left(\frac{Ca}{S}\right)}^{\gamma }}$$

with the basal release rate *r_basal_* and the maximum release rate *r_max_*. The set-point *S* is the Ca^2+^concentration, where the slope of the release function is steepest. It is close to the individual baseline in plasma Ca^2+^concentrations. Therefore, tiny changes from the baseline in plasma Ca^2+^concentrations elicit significant changes in plasma PTH concentrations.

**Figure 1:**
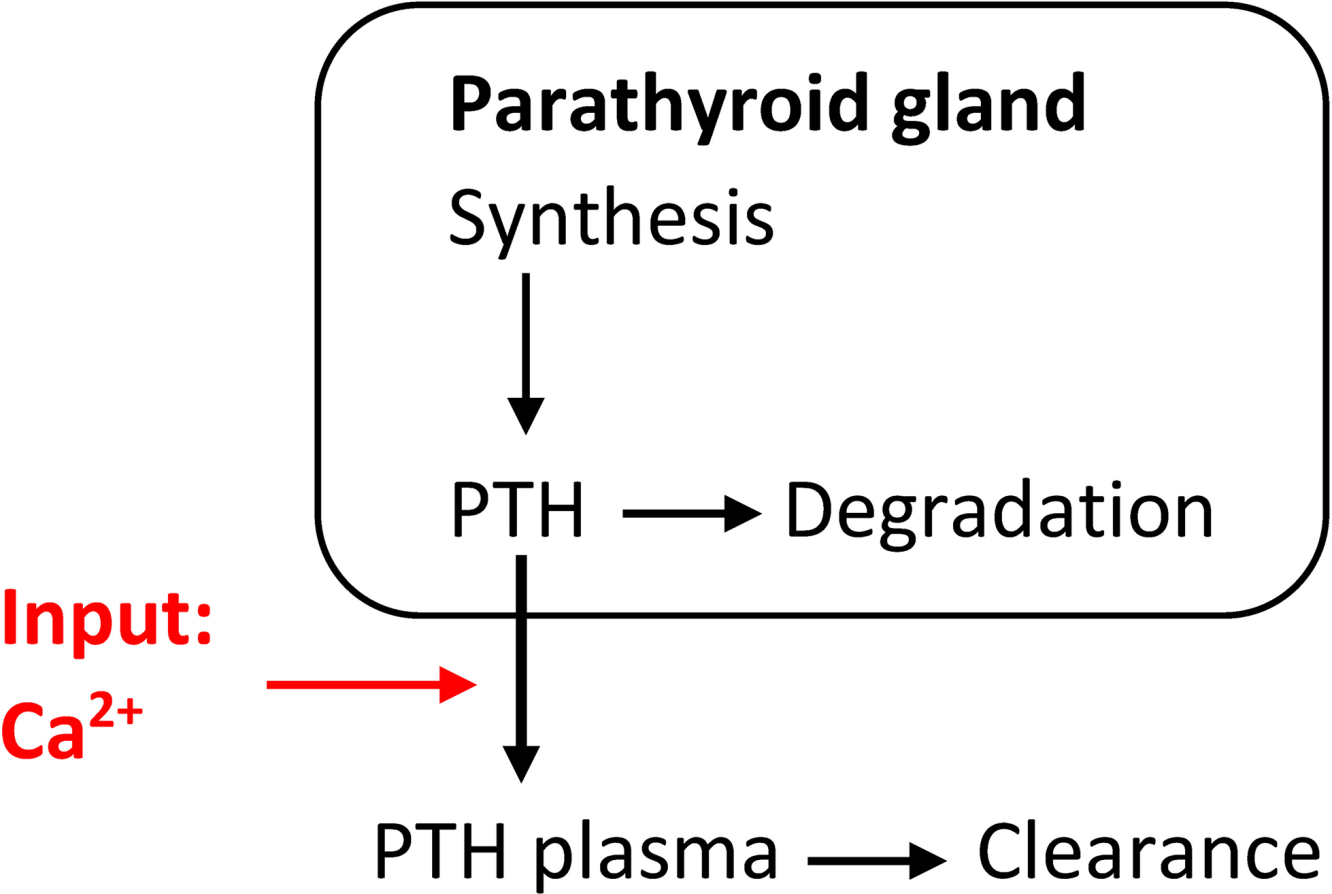
Simple model of the relationship between PTH release and changes in Ca^2+^concentrations able to at least partly explain hysteresis.


This simplistic PTH model is part of some calcium homeostasis models, e.g., (28). However, this model is not complex enough to reflect hysteresis, i.e., the different relationship between Ca^2+^and PTH concentration depending on whether Ca^2+^is increasing or decreasing (29, 30). A dynamic model able to (at least partly) explain this phenomenon takes the PTG’s ability to store synthesized PTH into account. This leads to a system of differential equations (**Figure 1**):


(3) $$\frac{dPTG}{dt}=k_{synth}-k_{rel}\left(Ca(t)\right)PTG(t)-k_{deg}PTG(t)$$

(4) $$\frac{dPTH}{dt}=k_{rel}\left(Ca(t)\right)PTG(t)-k_{cl}PTH(t)$$

*k_synth_* is the mean production rate, *k_deg_* the intracellular degradation rate while *k_cl_* is the plasma clearance rate. The Ca^2+^-dependent PTH release function *k_rel_* has the typical sigmoidal shape (Equ. 2). of the release function is steepest. At steady state, we get the following relationships:


(5) $$PTG^{*}=\frac{k_{synth}}{k_{rel}\left(Ca\right)+k_{deg}}$$

(6) $$PTH^{*}=\frac{k_{rel}\left(Ca\right)PTG^{*}}{k_{cl}}$$

We know the half-life of PTH in circulation. It is reported to be around 2-3 minutes (11, 31). The half-life of PTH in the parathyroid cells is significantly greater.

We can estimate model parameters from so-called calcemic clamp tests. In these tests, plasma Ca^2+^concentration is either decreased by intravenously administered sodium citrate or increased by intravenously administered calcium gluconate (11). If the time steps between two subsequently measured Ca^2+ ^and PTH concentrations are sufficiently small, e.g., 5 minutes for rapid changes in concentrations and 10 minutes else, the parameters can be estimated from these experiments [e.g., (32)].

Individualized model parameters would mainly inform us about the apparent individual set-point (11) associated with the density of calcium sensing receptors (33). A shift of the set-point to higher Ca^2+^concentrations indicates the loss in CaSR sensitivity or density or both. Assuming a narrow distribution of the set-point in the healthy population, we can estimate what effect, e.g., a calcimimetic or calcitriol therapy must achieve to reduce the PTH secretion rate. However, the model parameter estimations and predictions are limited because the mass of the PTG is not taken directly into account, and the number of chief cells affects the limits of PTH production (34). We can simulate the effect of an intervention and the subsequent shift of the apparent set-point to lower Ca^2+^concentrations on PTH secretion rates. However, options to predict the effect of an intervention on the PTH plasma concentrations are limited.

### 2.2 Coarse-Grain Model Incorporating Long Term Effects

**Figure 2:**
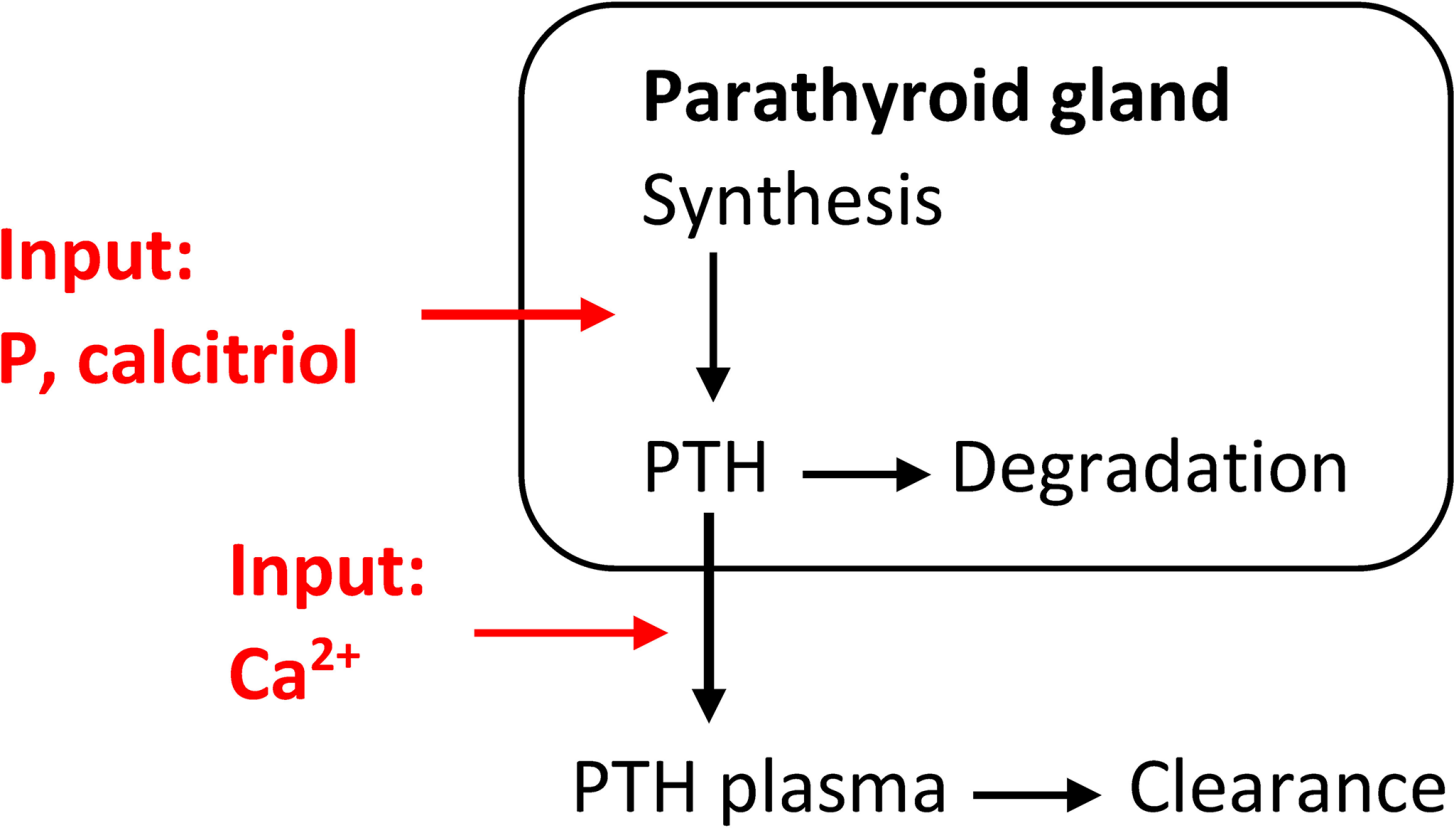
Coarse-grain model of the relationship between PTH release and changes in Ca^2+^concentrations including a phenomenological model for the synthesis rate of PTH. This model is able to simulate secondary hyperparathyroidism.


Additionally to short-term effects, there are long-term changes in CasR sensitivity, intracellular degradation rate, synthesis rate, and PTG proliferation rate. We could take all these effects into account in a coarse-grain model, i.e., we describe the changes in PTG indirectly *via* the mass of the PTG, e.g., (35). Again, the release rate is described through a sigmoidal relationship, but PTH synthesis is influenced by PTG mass. However, we can take other influences, such as the positive feedback loop between calcitriol and CaSR, into account (7). We can estimate generic model parameters from literature data, e.g., (13, 29, 36), or calcemic clamp experiments as described above. This model approach allows us to reflect secondary hyperparathyroidism *via* adaptation of PTG mass. For example, Peterson and Riggs (35) used an inverse multiplicative sigmoidal expression for the growth in PTG mass as a function of plasma calcitriol concentrations (*D*) (6). Additionally, we could consider a second multiplicative sigmoidal expression to reflect the observed increase in PTG mass due to increased plasma phosphate concentrations (*P*) (22, 23, 37) as described in, e.g., (38, 39) (**Figure 2**):


(7) $$\frac{dPTG}{dt}=k_{synth}\left(cal,P\right)-k_{rel}\left(Ca(t)\right)PTG(t)-k_{deg}PTG(t)$$

(8) $$\frac{dPTH}{dt}=k_{rel}\left(Ca(t)\right)PTG(t)-k_{cl}PTH(t)$$

The synthesis rate can be modeled by multiplicative sigmoidal expressions:


(9) $$k_{synth}(D,P)=k_{synth}^{basal}H_{P,synth}^{+},(P)H_{D,synth}^{-}(D)$$

where the functions $H_{P,synth}^{+}$ and $H_{D,synth}^{-}$ are sigmoidal functions, e.g. of the form


(10) $$H_{a,b}^{+}(x)=\alpha_{a,b}+\left(\beta_{a,b}-\alpha_{a,b}\right)\frac{x^{\gamma_{a,b}}}{\delta_{a,b}^{\gamma_{a,b}}+x^{\gamma_{a,b}}}$$

(11) $$H_{a,b}^{-}(x)=\alpha_{a,b}-\left(\alpha_{a,b}-\beta_{a,b}\right)\frac{x^{\gamma_{a,b}}}{\delta_{a,b}^{\gamma_{a,b}}+x^{\gamma_{a,b}}}$$

This type of model has a clear advantage over the more simplistic ones when it comes to calcium and phosphate homeostasis models, e.g., (35, 38, 39). We can take the effect of the growing PTG mass on the limits of PTH production rate into account (34). Moreover, due to the multiplicative sigmoidal function of calcitriol, we can indirectly reflect the feedback loop between the VDR and CaSR on the density of the calcium-sensitive receptors (33). The same is true for high phosphate levels increasing the PTG mass. This way, secondary hyperparathyroidism can be simulated, and the simulations predict adequate long-term changes in PTH levels (35). Moreover, we can use this type of model for *in-silico* generation of new research hypotheses. However, there are some shortcomings of this approach. The coarse-grain model is not mechanistic, i.e., the changes in PTG mass are incorporated by a phenomenological model. In the case of corrected phosphate concentrations and calcitriol concentrations, PTG mass will decline. The decline is in contrast to clinical studies where the decrease has been observed in some studies, e.g., (40), but not in others, e.g., (41). Therefore, this approach might be too simplified for correct predictions of the state of an individual PTG. While predictions on the cohort level are feasible (38), the estimation of individualized parameters of the sigmoidal functions and their validation is not possible without further constraints. Specifically, the synthesis rate and the parameters of the multiplicative sigmoidal functions are non-identifiable as changes in one parameter can be compensated by changes in other parameters.

### 2.3 Mechanistic Model for Secondary Hyperparathyroidism

Parathyroid gland biology is significantly altered in patients with secondary hyperparathyroidism. The base values differ considerably between healthy volunteers and secondary hyperparathyroidism patients. Also, the dynamics of the responses of the PTG to changes in Ca^2+^concentrations differ significantly (42). As discussed before, simplified models can predict some of the behaviors of interest. However, to fully grasp secondary hyperparathyroidism, a detailed mechanistic model reflecting the state of the PTG is necessary. Combined with patient-specific model parameters, this approach allows us to predict the efficacy of different therapies.

**Figure 3:**
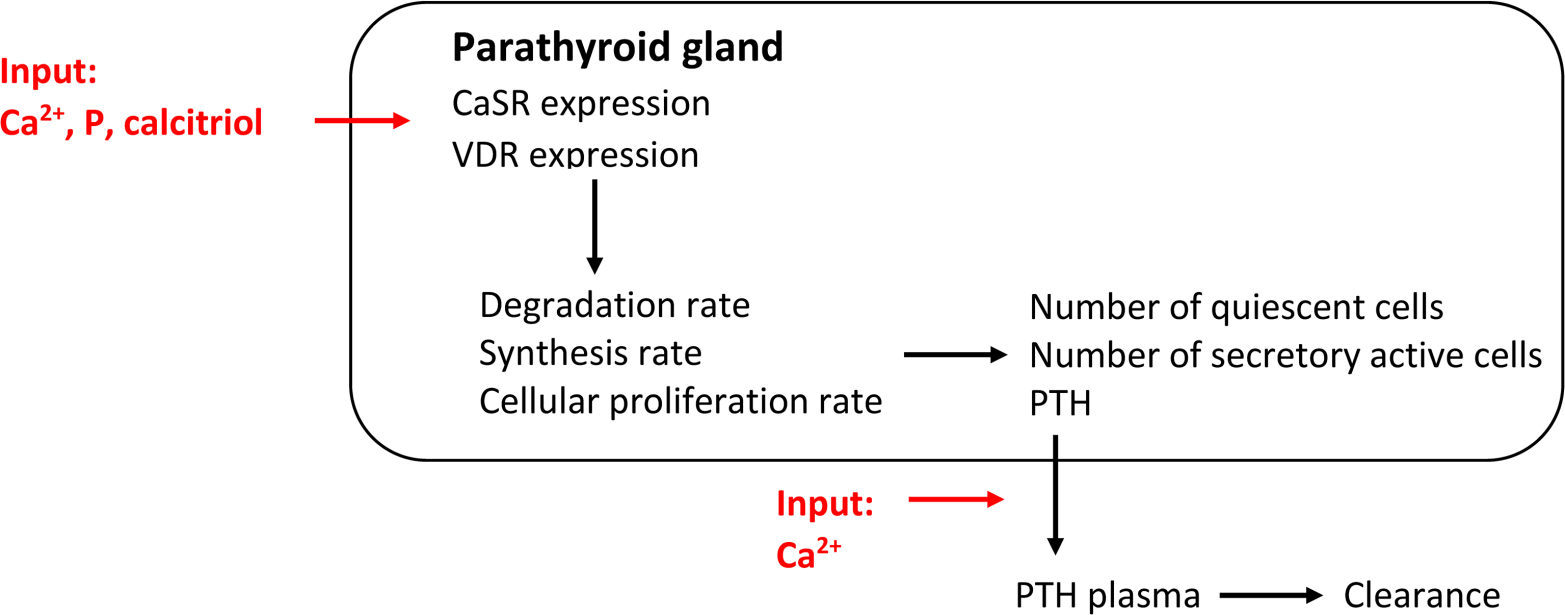
Comprehensive model of PTG biology governed by CaSR and VDR expression. This mechanistic models captures all PTG adaptation mechanisms.

A mechanistic model of PTG biology takes the signaling of the CaSR and VDR and the influence of critical parameters on the receptors into account. Thereby, the adaptation of PTH synthesis rate, intracellular degradation rate, and PTG cell proliferation rate can be properly accounted for. While such mathematical models are complex, a mechanistic model of PTG biology was presented in (43) where the authors took the physiological principles governing the CaSR signaling cascade into account (**Figure 3**). Specifically, if one or more critical parameters, i.e., Ca^2+^, phosphate, and calcitriol concentrations, are not in their optimal range for a critical amount of time, CaSR signaling is altered. It results in adaptations of PTH release rate, intracellular degradation rate, synthesis rate, and PTG cell proliferation rate. The alteration in signaling also changes CaSR density over time. The deviation from the optimal range is described *via* stimulus function. All adaptation effects but hyperplasia are reversible. Since the apoptosis rate is supposedly constant (17, 18), the mass of the PTG will not decrease.

**Figure 4:**
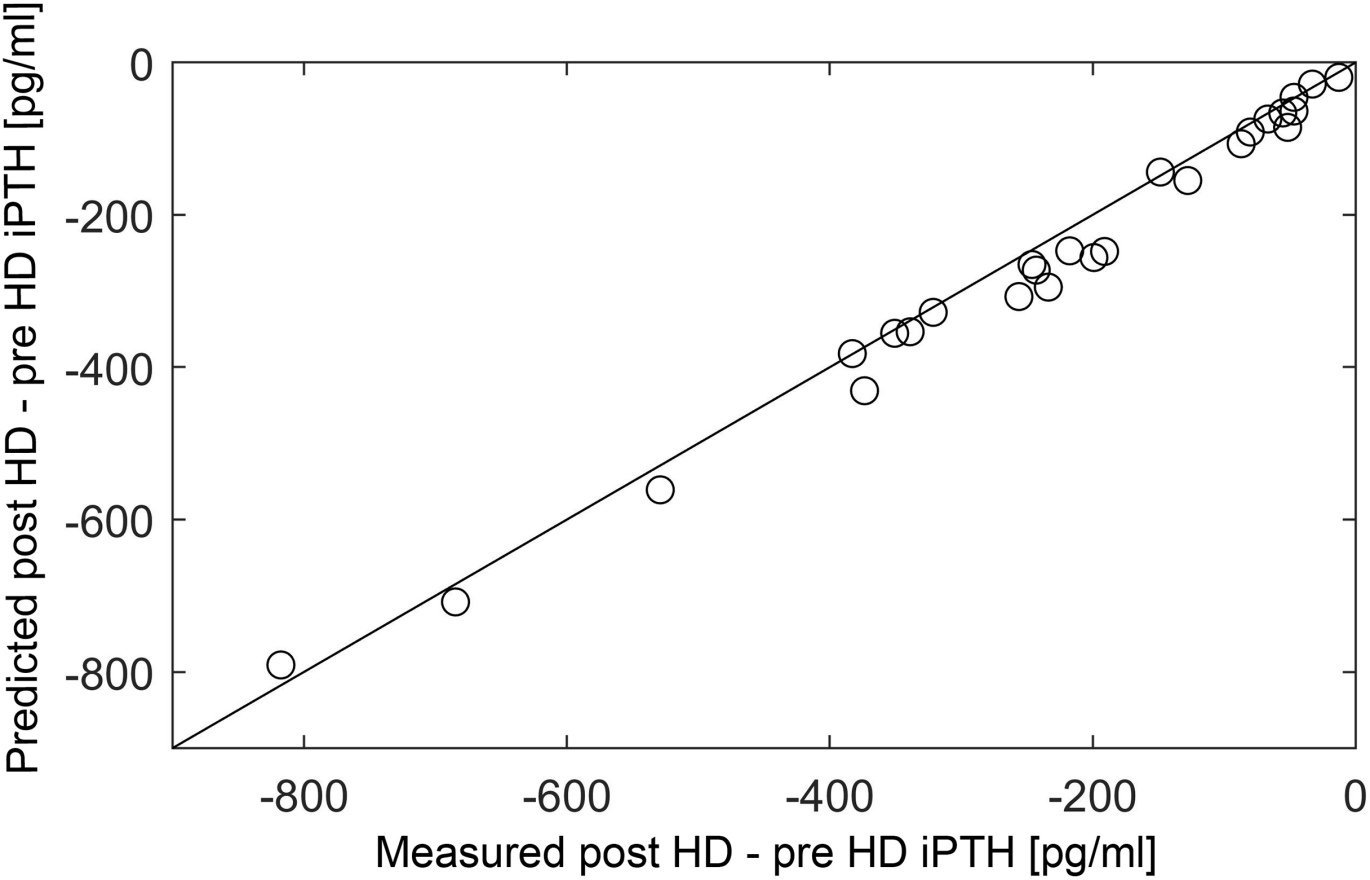
Correlation between measured and predicted post-minus-pre HD iPTH (n = 26). Measured (x-axis) and predicted (y-axis) peridialytic PTH change (r = 0.984, p < 0.001). The peridialytic change was calculated as post-HD iPTH minus pre-HD iPTH levels. Reproduced from (44); Creative Commons BY 4.0; published by Frontiers (2021).

In (44), we could show that we can estimate the model parameters for individual patients based on physiological principles and readily available clinical data, i.e., dialysis vintage, six months routinely measured Ca^2+^concentration, and phosphate concentration, information of calcitriol therapy. We could predict short-term PTH kinetics and trends in long-term PTH concentrations with the personalized parameters. Short-term PTH kinetics was assessed during a single dialysis session with a dialysate Ca concentration of 1.75 mmol/l to attain a positive dialysate-to-plasma Ca^2+^gradient (**Figure 4**). Long-term kinetics was assessed during a six months follow-up period.

With the personalized model coupled with pharamcokinetic and pharmacodynamic models, we can predict the efficacy of different treatment options such as phosphate binders, calcitriol therapy, or the administration of calcimimetics (45), and thereby, provide an informed recommendation for the treatment strategy.

## 3 Discussion

Adequate management of sHPT is of utmost importance for precision medicine approaches in the dialysis population. However, individual prediction of sHPT disease courses has not been accomplished in clinical practice yet. Population-based risk assessment does not necessarily reflect individual patients’ disease course. For example, incident HD patients with mildly elevated iPTH levels (suggestive of early-stage sHPT and good treatment response on a population level) might not respond to PTH-lowering treatments. In contrast, some maintenance dialysis patients with persistently high iPTH levels (suggestive of advanced sHPT and poor treatment response on a population level) might respond well to pharmacologic treatment (25). Mathematical models of PTG biology, thus, can help to predict long-term PTH dynamics better and thus, allow for better prognosis and personalized treatment approaches.

In general, mathematical models proved extremely useful for studying complex physiological processes. They allow us to study subsystems while keeping different influences constant. They enable the generation of new working hypotheses and quantitative and qualitative predictions that can be compared with clinical or experimental data. An example would be the prediction of calcium buffer capacity during hemodialysis (46) which was confirmed by clinical data on the population level (47). In recent years, mathematical models have become an essential tool in personalized medicine. An already established example in hemodialysis patients would be the optimized administration of erythropoiesis-stimulating agents based on a mathematical model for erythropoiesis and individualization of the model parameters (48, 49). Here, we discuss the different approaches to mathematical models of PTG biology. Significant heterogeneity of clinical phenotypes partly based on individual variations in PTG cell proliferation pattern and the expression and genetics of VDR and CaSR call for a personalized approach.

Different models are trying to describe and explain PTH secretion rate or PTG biology. Simpler models can accurately predict the immediate response of PTH to changes in Ca^2+^concentrations. Parameters such as the apparent set-point in the sigmoidal function of Ca^2+^describing the PTH release rate can be estimated on the individual level, e.g., (32, 50). However, other predictions are limited since the complex adaptation mechanisms of the PTG leading eventually to secondary hyperparathyroidism cannot be taken into account. More complex but phenomenological models can be used to simulate the development of sHPT (35). However, estimation of personalized model parameters is not feasible without hypothesis-based assumptions. Moreover, complex and inter-individual responses cannot be predicted since the corresponding physiological mechanisms are not considered.

While simplified models might not be applicable in clinical practice, more sophisticated models capturing PTG biology allow clinicians to predict long-term iPTH dynamics on a patient-level utilizing readily available clinical parameters only (HD vintage, iPTH history, phosphate, and iCa values, as well as information about calcitriol therapy). The most complex model is based on physiological principles and takes the CaSR and VDR into account (44). While this model is hard to read and implement, it has been shown that predictions based on personalized model parameters accurately predict peridialytic PTH responses to calcium loading and PTH trends over six months. Combined with pharmacokinetics/pharmacodynamics models, the mechanistic models can provide robust predictions of treatment efficacy. Given that treatment decisions in sHPT management should be based on trends rather than single lab values, this long-term prediction of iPTH dynamics might directly influence treatment decisions and help clinicians achieve PTH target levels earlier and more frequently. Furthermore, these sophisticated PTG models can help predict iPTH control in response to therapeutic phosphate lowering in calcimimetic-naïve patients.

Future model iterations should integrate calcimimetic drug effects and the pharmacokinetic model of calcitriol therapy to optimize the personalized management of sHPT. Since phosphate is a significant driver of sHPT, integrating a complex mathematical model of PTG biology in a comprehensive calcium and phosphate homeostasis model to capture the effect of phosphate binders will be the focus of future studies. There have

The main advantage of model predictions is the complete freedom in selecting treatment options. In addition, the models provide the unique opportunity to choose the time frame we are interested in, e.g., six months, and keep all other parameters constant. Thereby, we can truly quantify the effect of a treatment. Thus, the *in-silico* trial enables us to reduce the dimensionality and the complexity.

In summary, a physiology-based mathematical model of PTG biology can predict short- and long-term iPTH levels in maintenance HD patients. The prediction power strongly depends on the complexity of the model and the data available for validation. The following steps are prospective studies to fully understand the use of this precision approach.

## Author Contributions

GS-T and MP: research idea and study design. GS-T, MP, and PK: manuscript preparation, drafting, and approval of the final version. All authors contributed to the article and approved the submitted version.

## Funding

The Austrian National Bank supported this work by providing MP with an educational research grant (OENB Jubiläumsfonds Project No. 18362). The funding institution had no role in study design, manuscript preparation, or decision to submit the manuscript for publication.

## Conflict of Interest

PK is an employee of the Renal Research Institute, a wholly owned subsidiary of Fresenius Medical Care. PK holds stock in Fresenius Medical Care. GS-T and PK are inventors on patents in the kidney space.

The remaining author declares that the research was conducted in the absence of any commercial or financial relationships that could be construed as a potential conflict of interest.

## Publisher’s Note

All claims expressed in this article are solely those of the authors and do not necessarily represent those of their affiliated organizations, or those of the publisher, the editors and the reviewers. Any product that may be evaluated in this article, or claim that may be made by its manufacturer, is not guaranteed or endorsed by the publisher.

## Footnotes

1 https://www.ncbi.nlm.nih.gov/pubmed
